# Vascular Imaging is the Only Reliable Method to Exclude Blunt Cerebrovascular Injury Post Hanging or Strangulation

**DOI:** 10.1002/wjs.12501

**Published:** 2025-02-04

**Authors:** Muhammad Zafar Khan, Howard Wain, Afroz Khan, Damian L. Clarke

**Affiliations:** ^1^ Department of Vascular & Endovascular Surgery Chris Hani Baragwanath Academic Hospital Johannesburg South Africa; ^2^ Department of Surgery University of the Witwatersrand Johannesburg South Africa; ^3^ Department of Surgery & Trauma Greys Hospital Pietermaritzburg South Africa; ^4^ Department of Surgery University of Kwa‐Zulu Natal Durban South Africa; ^5^ Department of Engineering University of Stellenbosch Stellenbosch South Africa

**Keywords:** hanging, stroke, vascular trauma

## Abstract

**Background:**

Strangulation and hanging injuries are a common form of self‐inflicted harm. The incidence of associated blunt cerebrovascular injury and its correlation with clinical findings remains unclear. Furthermore, the decision to image and the choice of imaging remains unresolved.

**Methods:**

A multicenter retrospective review of a prospectively collected database was performed of all patients who presented with a hanging or strangulation injury. All patients underwent a CT angiogram. Inferential and descriptive statistics were performed. Patient demographics, clinical data, laboratory results, and imaging findings were reviewed.

**Results:**

Over an 11‐year period (2013–2023), 194 patients presented with hanging or strangulation type injuries. The median age was 28.7 years, and 24% were female. All patients underwent a CT angiogram with only 9 patients (4.6%) having a positive finding. There was no correlation between positive findings on CT angiogram and any clinical or laboratory finding. The in‐hospital mortality rate was 1%, none of whom had an associated vascular injury.

**Conclusion:**

The incidence of vascular injury in strangulation or hanging injuries is 4.6%. In light of the reported incidence of stroke in blunt carotid injury (23%–30%) when left untreated, it is imperative that these injuries are found and treated. Based on the above data, there are no clinical or lab indicators (or combination thereof) that can be safely used to exclude an injury and avoid imaging. Thus, is it advised that all patients are imaged with a CT angiogram.

## Graphical abstract

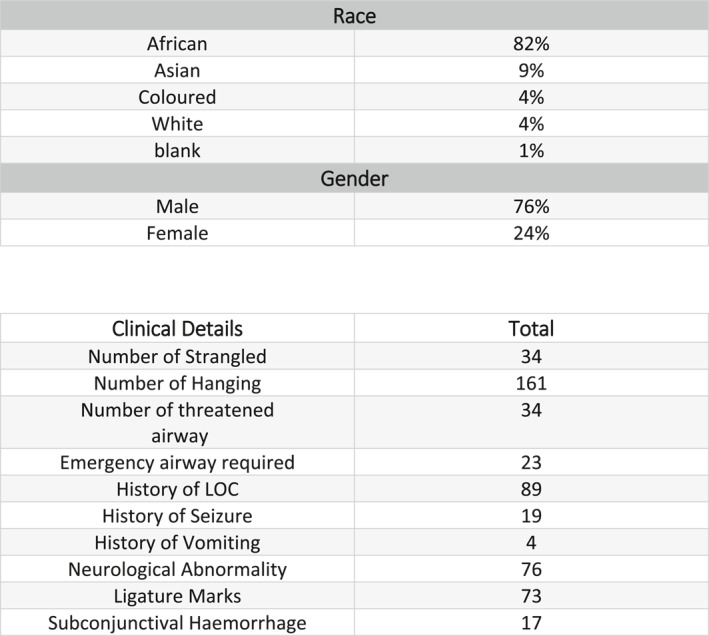

## 1 Introduction [1, 2, 3, 4, 5, 6, 7, 8, 9, 10, 11, 12, 13, 14]

Hanging and strangulation injuries are a common form of self‐inflicted harm. Although there have been recent publications on the topic in the last decade, the ideal management algorithm of these patients remains unclear. Initial management of a hanging injury must follow the advanced trauma life support (ATLS) course approach of primary survey with a focus on airway management and cervical spine immobilization. Once this has been achieved, most authors advocate imaging of the neck structures, usually in the form of a contrast‐enhanced computed tomography (CT) scan of the head and the neck. This will assess intracranial infarction or hemorrhage, cervical spine injury, and aerodigestive tract and extracranial blunt cerebra‐vascular injury (BCVI) [1, 2, 3, 4, 5, 6].

BCVI refers to injuries of the extracranial carotid and vertebral arteries that occur in the absence of penetrating trauma. BCVI is seen in 1%–2% of all admitted trauma patients and in up to 9% of patients with severe traumatic brain injury. BCVI is strongly associated with cervical spine, base of the skull, and facial fractures. The mechanism of BCVI starts with a series of flexion, extension, and rotatory forces that stretch and impinge on the artery. This leads to intimal injury, which may subsequently evolve to thrombosis, occlusion, or pseudoaneurysm formation. Acute stroke may occur due to thrombosis or embolization. The historic incidence of stroke in the pre‐screening era was 50%, where the first detection of a BCVI was a stroke. The current incidence is 1%–26%. This is more common with carotid than vertebral artery injury. Stroke risk has a linear relationship with the grade of BCVI based on the BIFFL grade, and the presence of stroke in BCVI is an independent risk factor for morbidity, mortality, and poor outcome (See Supporting Information S1: Appendix A BIFFL Grading System). [4, 6, 7, 8, 9].

The early identification and management of BCVI (by antithrombotic and/or endovascular therapy) has been proven to reduce stroke risks from 26% to < 1%. The 2012 expanded Denver screening criteria is a well‐established screening tool to determine indication for CT angiography in trauma to detect BCVI. It is currently also the preferred tool in pediatric populations over the Utah screening criteria where a retrospective review found the 2012 expanded Denver screening criteria to be superior (See Supporting Information S1: Appendix A for Criteria). One of the limitations of the Denver screening criteria is an absence of commentary on patients with hanging or strangulation injuries. To date, there have been no publications that have found a correlation between clinical findings, such as ligature marks, subconjunctival hemorrhage, and a positive finding on CT angiogram. This project aims to review the yield of routine CT scan of the head and the neck post hanging and sets out to interrogate in detail the incidence of blunt cerebrovascular injury (BCVI) associated with hanging. Furthermore, we aimed to assess for any clinical features that could reliably exclude BCVI in these patients [4, 10, 11, 12, 13, 14].

## 2 Clinical Setting

Pietermaritzburg is the capital city of the KwaZulu‐Natal Province in South Africa. The department of surgery at Greys and Edendale Hospitals provides tertiary surgical and trauma service to the city of Pietermaritzburg and the western third of the province. This comprises a population of over three million people. There are other major urban conurbations in the northern parts of the province around the towns of Ladysmith and Newcastle. The rest of the area is largely rural [4].

All trauma patients are seen, resuscitated, and imaged by the Emergency Medicine Department and referred on to the trauma unit thereafter for definitive care. Patients with associated vascular trauma are managed in conjunction with a consultant vascular surgeon in the metropolitan. All surgical procedures are performed within the metropolitan by trauma and vascular surgery consultants. All patients admitted are recorded on a hybrid electronic database. Ethics approval for the maintenance of our registry and for this study was formally approved by the Biomedical Research Ethics Committee of the University of KwaZulu‐Natal (Reference number: BCA 207/09 and BCA 221/13).

## 3 Methodology

All patients who had sustained a hanging or strangulation injury were identified using a word search of the database. Patient demographics, mechanism of injury, admission vital signs, clinical findings, lab and imaging findings, management strategy (operative or nonoperative), complications, and 30‐day inpatient mortality rates were extracted.

## 4 Definitions

- Hanging in forensic medicine is defined as asphyxia and neuronal death caused by the suspension of the body by a ligature, which encircles the neck, with the constricting force being the weight of the body.
- Strangulation in forensic medicine is defined as asphyxia and neuronal death due to any extrinsic compression of the airway or vascular structures, which impedes circulation or function.
- Blunt cerebrovascular injury is defined as injuries of the extracranial carotid and vertebral arteries that occur in the absence of penetrating trauma.

## 5 Statistics

Descriptive statistics were calculated for age, demographics, biochemical, clinical, and physiologic parameters as well as in‐hospital mortality. Central tendency was expressed as medians and interquartile ratios. Clinical findings were expressed as frequencies and percentages of the total sample.

## 6 Results

During the 11‐year period, January 2013–December 2023, under review, a total of 194 patients were admitted with a hanging injury (161) or strangulation injury (34). Of these, 2 patients demised in the hospital (1%). Neither of the deaths was related to BCVI. The median age of patients was 28.7 years with a male to female ratio of 3:1 (male 76% and female 24%). Table 1 summarizes the demographics, mechanism of injury, and presenting clinical signs of all patients with hanging or strangulation injury.

**TABLE 1:** Demographic details and admission findings of the entire sample.

Race	
African	82%
Asian	9%
Colored	4%
White	4%
Blank	1%
Gender	
Male	76%
Female	24%

Clinical details	Total
Number of strangled	34
Number of hanging	161
Number of threatened airway	34
Emergency airway required	23
History of LOC	89
History of seizure	19
History of vomiting	4
Neurological abnormality	76
Ligature marks	73
Subconjunctival hemorrhage	17

### 6.1 Demographics

Demographic analysis revealed a predominance of African patients at 82% (159), followed by Asian/Indian 9% (17), Caucasians 4% (8), mixed race 4% (8), and unspecified 1% (2).

### 6.2 Clinical Findings

A total of 17.5% (34) presented with a threatened airway with 12% (23) requiring an emergency airway intervention by endotracheal intubation. A history of loss of consciousness (LOC) was present in 46% (89), seizures in 9% (19), vomiting in 2% (4), positive neurological abnormality in 39% (76), ligature marks in 37% (73), and subconjunctival hemorrhage in 8% (17). There was no correlation noted between any of the above clinical criteria and a positive vascular imaging finding on CTA Angiogram.

### 6.3 Imaging Findings

All patients underwent imaging either by CT angiogram (97% (188)) or carotid duplex ultrasound study (3% (6)). A total of 4.6% (9) of patients had an associated vascular injury. Common carotid injuries occurred in 7 patients, including one patient with bilateral injuries. Vertebral artery injuries occurred in 2 cases. Table 2 summarizes the associated clinical findings, and Supporting Information S2: Appendix B provides details on the BIFFL score, intervention, and outcome. There was no correlation between patients with a positive vascular injury on angiogram and any clinical finding such as airway injury, LOC, seizures, vomiting, ligature marks, or subconjunctival hemorrhage.

**TABLE 2:** Summary of all cases with positive vascular injury on imaging.

Clinical findings	CT positive finding %		CT negative finding %	Total
Strangled	14.7		85.3	34
Hanging	3.1		96.9	161
Trachea central	4.6		95.4	193
Threatened airway	3		97	34
Emergency airway required	4.3		95.7	23
Resp: None	4.3		95.7	162
Resp: PPV	4		96	25
Resp: FMO2	16.6		83.4	6
History of mechanism	4.6		95.4	194
History of LOC	2.2		97.8	89
History of seizure	5.2		94.8	19
History of vomiting	0		100	4
Neurological abnormality	1.3		98.7	76
Ligature marks	4.1		95.9	73
Subconjunctival hemorrhage	5.8		94.2	17

### 6.4 Outcome

A total of 9 patients (4.6%) were found to have a positive vascular injury on CT angiogram. All patients were managed nonoperatively with inpatient antithrombotic therapy with a single antiplatelet agent (aspirin) with a 0% inpatient stroke rate and a 0% mortality rate. No patients required endovascular or open surgery. Patients were admitted from five to 7 days for clinical monitoring and then discharged on aspirin for 1 month. All patients were referred for psychological and psychiatric assessment. All patients were given a 30‐day clinical outpatient follow‐up date, and those with a pseudoaneurysm were scheduled for a 30‐day repeat CT angiogram. Unfortunately, however, no patient returned for follow‐up at 30 days. This is a limitation of the study.

## 7 Discussion [1, 4, 8, 9, 14, 15, 16, 17, 18, 19, 20, 21, 22, 23, 24, 25]

The ATLS approach to a hanging victim focuses on establishing an adequate airway and ensuring adequate cerebral perfusion. Once this has been achieved, detailed imaging is necessary. There are several areas of clinical concern, and these include the central nervous system, the airway, and the vasculature. There are a variety of imaging modalities available. CT angiogram remains the most practical imaging tool available; it can assess the central nervous system, as well as the soft and bony tissue in the neck and allows for detailed angiography. CTA has a reported sensitivity of 66%–97% for detecting an injury to the vasculature of the neck. This wide range may be explained by the level of CTA film slices (16, 32, and 64). There also remains a risk of false positivity with CT angiography due to associated vessel spasm and artefact. Magnetic resonance angiography has an equal sensitivity to CT angiography but is more time‐consuming and has limited availability. The additional advantage of MRA is to detect early cerebral ischemia. Catheter‐directed angiography has a sensitivity of 90%–95% and remains the historic gold standard investigation providing a detailed luminogram with information on flow dynamics. However, due to its invasive nature and associated periprocedural stroke risk of 1.3% (arterial dissection, thrombosis, and embolization), is reserved for cases of diagnostic doubt or for associated endovascular intervention. Carotid duplex scanning (DUS) is a useful, noninvasive tool with excellent evaluation of the vessel wall and intima and a sensitivity of 38%–86%. However, it is limited by interobserver variability and a poor acoustic window above the angle of the mandible [1, 4, 8, 9, 14].

Selecting patients for imaging is controversial. The 2012 expanded Denver screening criteria advocates a selective approach and recommends CT angiography in cases of near hanging with associated hypoxic brain injury and in patients with a clothesline or seat belt abrasion with associated swelling or altered mental status. In our setting, we routinely image all patients who survive a hanging or strangulation. Our incidence of hanging‐related BCVI of 4.6% correlates with the reported 5% in the current body of literature available. This is a relatively high yield from CT scan, and this justifies a policy of routine CT scan in all patients who sustain a hanging‐related injury is reasonable [1, 4, 8, 9, 14].

BCVI if left untreated, is an associated 1%–26% risk of stroke. Given the limited correlation in our data between BCVI and clinical parameters such as airway injury, seizures, LOC, neurology, ligature marks, and subconjunctival hemorrhage, we recommend all patients undergo imaging to detect a possible BCVI that may be treated to dramatically reduce stroke risk and morbidity. To date, there is no consensus or high‐quality data in the literature on the ideal management of all BCVIs. Most societal guidelines have recommended antithrombotic therapy for all patients with BIFFL 1, 2, and 4 injuries with a proven reduction in stroke risk from 26% to 0%–5%. The choice of antithrombotic therapy is not defined, but may include antiplatelet therapy or low molecular weight heparin. The current evidence, based on multiple small heterogenous cohorts, shows no statistical difference in stroke reduction between antiplatelet and anticoagulant therapy with no major difference in bleeding risk. Endovascular therapy is reserved for select patients with BIFFL grade 2 injuries and all patients with BIFFL grade 3 injuries. Open surgery remains an alternative solution in BIFFL grade 5 injuries and select cases of hemodynamic instability, easily accessible injury (e.g. zone II), or contraindication to long‐term antithrombotic therapy [15, 16, 17, 18, 19, 20, 21, 22, 23, 24, 25].

Given the limited data in the literature on this specific group of blunt carotid artery injuries with mechanisms of hanging or strangulation, future multicentered studies of this specific injury population subgroup is needed.

## 8 Conclusion [4, 6, 7, 8, 10]

Our data are in keeping with the international literature and suggests a relatively high rate of BCVI post hanging of just under 5%. This high yield justifies a nonselective approach to imaging in these patients. We recommend that all patients with a history of hanging or strangulation mechanism injury undergo a CT angiogram, as there are no clinical features that reliably exclude BCVI.

## Author Contributions

**Muhammad Zafar Khan:** conceptualization, formal analysis, investigation, methodology, writing–original draft, writing–review & editing. **Howard Wain:** conceptualization, data curation, formal analysis, investigation, methodology, writing–review & editing. **Afroz Khan:** formal analysis. **Damian L. Clarke:** conceptualization, writing–review & editing.

## Conflicts of Interest

The authors declare no conflicts of interest.

## Supporting information

Supporting Information S1

Supporting Information S2
