# Supplementary material for: Vascular Imaging is the Only Reliable Method to Exclude Blunt Cerebrovascular Injury Post Hanging or Strangulation
Source: World J Surg. 2025 Feb 4;49(3):752–6. doi: 10.1002/wjs.12501 (PMC11903248; doi:10.1002/wjs.12501)
Supplement: Supplementary file 1 — Supporting Information S1 [file WJS-49-752-s002.docx]

**Biffl grading system:**

| Injury grade | Description | Stroke rate (%) | Mortality rate (%) | Management |
| --- | --- | --- | --- | --- |
| I | Luminal irregularity or dissection with | 3 | 11 | Anticoagulation + carotid stenting (if progression of grade I/II injury/ enlarging pseudo-aneurysm) |
| II | Dissection or intramural haematoma with >25% luminal narrowing or intimal flap | 11 | 11 | As for grade I |
| III | Pseudo-aneurysm | 33 | 11 | As for grade I |
| IV | Occlusion | 44 | 22 | Anticoagulation |
| V | Transection with free extravasation | 100 | 100 | Open repair |

**Expanded Dever Screening criteria:**

| Signs and symptoms | Riks factors:  *High-energy transfer mechanism plus any of the following 4:* |
| --- | --- |
| Potential arterial haemorrhage from the neck, nose, or mouth | Le Fort II or III displaced midface fracture |
| Cervical bruit in patients <50 years of age | Mandible fracture |
| Expanding cervical hematoma | Complex skull fracture (e.g., involving frontal bone and orbit) |
| Focal neurologic deficit (transient ischemic attack, hemiparesis, vertebrobasilar symptoms, Horner syndrome) | Base of skull fracture (sphenoid, petrous temporal, clivus, and occipital condyle fractures) |
| Neurologic deficit incongruous with head CT findings | Scalp degloving |
| Stroke on CT or MRI | Cervical spine fracture, subluxation, or ligamentous injury at any level |
|  | Severe traumatic brain injury with Glasgow coma scale <6 |
|  | Near hanging with hypoxic-ischemic (anoxic) brain injury |
|  | Clothesline type injury or seat belt abrasion with significant swelling, pain, or altered mental status |
|  | Traumatic brain injury with thoracic injuries |
|  | Upper rib fractures |
|  | Thoracic vascular injuries |
|  | Blunt cardiac rupture |
