# Supplementary material for: Vascular Imaging is the Only Reliable Method to Exclude Blunt Cerebrovascular Injury Post Hanging or Strangulation
Source: World J Surg. 2025 Feb 4;49(3):752–6. doi: 10.1002/wjs.12501 (PMC11903248; doi:10.1002/wjs.12501)
Supplement: Supplementary file 2 — Supporting Information S2 [file WJS-49-752-s001.docx]

Appendix B: BIFFL Grading of cases, intervention and Outcomes

| **BIFFL Grade of Injury** | **Artery Involved** | **Intervention** | **Outcome** |
| --- | --- | --- | --- |
| B2, B4 | Common Carotid (bilaterally) | Anti-thrombotic Therapy | Survived |
| B4 | Common Carotid | Anti-thrombotic Therapy | Survived |
| B1 | Common Carotid | Anti-thrombotic Therapy | Survived |
| B4 | Common Carotid | Anti-thrombotic Therapy | Survived |
| B2 | Common Carotid | Anti-thrombotic Therapy | Survived |
| B4 | Vertebral Artery V1 | Anti-thrombotic Therapy | Survived |
| B2 | Common Carotid | Anti-thrombotic Therapy | Survived |
| B2 | Vertebral Artery V1 | Anti-thrombotic Therapy | Survived |
| B1 | Common Carotid | Anti-thrombotic Therapy | Survived |
